# Comparative Proteomic Analysis Reveals Key Proteins Linked to the Accumulation of Soluble Sugars and Organic Acids in the Mature Fruits of the Wild *Malus* Species

**DOI:** 10.3390/plants8110488

**Published:** 2019-11-11

**Authors:** Baiquan Ma, Yuduan Ding, Cuiying Li, Mingjun Li, Fengwang Ma, Yangyang Yuan

**Affiliations:** State Key Laboratory of Crop Stress Biology for Arid Areas/Shaanxi Key Laboratory of Apple, College of Horticulture, Northwest A&F University, Yangling 712100, Shaanxi, China; bqma87@nwsuaf.edu.cn (B.M.); dingyuduan@nwafu.edu.cn (Y.D.); lcy1262@nwsuaf.edu.cn (C.L.); limingjun@nwsuaf.edu.cn (M.L.)

**Keywords:** apple, soluble sugars, organic acids, proteomics, parallel reaction monitoring

## Abstract

Soluble sugars and organic acids are the main determinants of fruit organoleptic quality. To investigate the genes responsible for the soluble sugar and organic acid contents of apple fruits, a label-free proteomic analysis involving liquid chromatography (LC)-mass spectrometry (MS)/MS was conducted with the fruits of two *Malus* species, *M. sargentii* and *M. niedzwetzkyana*, which exhibit significant differences in soluble sugar and organic acid contents. A total of 13,036 unique peptides and 1,079 differentially-expressed proteins were identified. To verify the LC-MS/MS results, five candidate proteins were further analyzed by parallel reaction monitoring. The results were consistent with the LC-MS/MS data, which confirmed the reliability of the LC-MS/MS analysis. The functional annotation of the differentially-expressed proteins, based on the gene ontology and Kyoto Encyclopedia of Genes and Genomes (KEGG) databases, revealed that they were mainly related to biological processes and cellular components. Additionally, the main enriched KEGG pathways were related to metabolic processes. Moreover, 31 proteins involved in soluble sugar metabolism, organic acid metabolism, and H^+^-transport were identified. The results of this study may be useful for the comprehensive characterization of the complex mechanism regulating apple fruit-soluble sugar and organic acid contents.

## 1. Introduction

Soluble sugars and organic acids are crucial determinants of fruit tastes, which combine with aromas to considerably influence the overall organoleptic quality of fruits [1,2]. Soluble sugars are the main source of carbon and energy in organisms, and are important for plant growth and development [3]. In fruits, soluble sugars mainly comprise sucrose, fructose, and glucose. Sucrose is commonly translocated to sink organs (carbon-demanding organs) via long-distance transport in phloem [4,5]. Three major organic acids (malic, citric, and tartaric acids) accumulate in most fruits, and their concentration in mature fruits is determined by the balance among acid synthesis, degradation, utilization, and compartmentalization in subcellular organelles [6,7]. The predominant organic acids in mature fruits vary among species. For example, malic acid is the major organic acid in apple, loquat, and pear [2,8,9,10], whereas citric acid is the predominant organic acid in citrus fruits [11].

Proteins are vital parts of living organisms and have diverse functions. Proteomics refers to the study of the proteins and includes investigations of protein expression levels, post-translational modifications, protein–protein interactions, and interactions between proteins and other biomolecules. Thus, proteomic analyses are important for studying biological systems [12]. In recent decades, approaches based on stable isotope labeling and label-free techniques have been used to conduct quantitative proteomic research [13]. Traditionally, methods involving two-dimensional electrophoresis coupled with mass spectrometry (MS), tandem MS (MS/MS), or liquid chromatography (LC)-MS/MS have been commonly used for quantitative proteomic investigations, including large-scale quantitative analyses [14]. To avoid the ion suppression effects of the MS signal for a particular peptide among co-eluting species, the LC-MS/MS approach with stable isotope-labeling techniques has been widely used for relative quantifications [15,16]. Although this strategy can accurately quantify protein abundances, labeling with a stable isotope is expensive and requires specific software and expertise to analyze the data. Moreover, some labeling procedures involve complex processes and yield artifacts [17,18]. As an alternative, label-free methods based on relative peptide peak intensities and protein abundances were recently developed. This label-free approach is applicable for any proteomic sample and does not require isotopes for quantification [19]. More recently, two targeted proteomic methods, selected reaction monitoring (SRM) and parallel reaction monitoring (PRM), have become prevalent. There may be several advantages to PRM over SRM, including greater specificity, higher tolerance for co-isolated background peptides/species, and no need for a preselection step [20,21,22].

Apple (*Malus × domestica* Borkh.) is one of the most important fruit crops in temperate regions, and its genome sequence has been released [23,24]. Apple fruits contain many components beneficial for human health, including soluble sugars, organic acids, and amino acids. The balance between soluble sugars and organic acids is responsible for the taste and flavor of apple fruits [25,26,27]. In apple fruits, the predominant soluble sugars are glucose, fructose, sucrose, and sorbitol, whereas the major organic acid is malic acid, which accounts for up to 90% of the total organic acid content [2,9,28]. A recent analysis of the organic acids in the mature fruits of 111 apple accessions (53 apple cultivars and 58 wild relatives) revealed that malic and citric acids are two principal organic acids in the *Malus* species [29]. The partitioning of soluble sugars and organic acids in apple fruits is a complex process that is initiated by the transport of photoassimilates (mainly sucrose and sorbitol) to sink tissues via phloem sieve elements. Almost all of the sorbitol and half of the sucrose are converted to fructose, and 80% of the total carbon flux goes through fructose in apple fruits [30]. Malic acid is mainly synthesized in the cytosol by the reactions catalyzed by phosphoenolpyruvate carboxylase (PEPC; E.C. 4.1.1.31) and malate dehydrogenase (MDH; E.C. 1.1.1.37), whereas it can be degraded via the decarboxylation catalyzed by MDH and the NADP-malic enzyme (ME; E.C. 1.1.1.40) [31]. Additionally, malic acid is mainly stored in vacuoles [32]. Citric acid is produced by the tricarboxylic acid (TCA) cycle in mitochondria. Specifically, it is synthesized by mitochondrial citrate synthase and degraded by aconitase and NAD-dependent isocitrate dehydrogenase (NAD-IDH), which are located in mitochondria and the cytosol, respectively [33,34]. The citric acid concentration in fruit cells is mainly determined by metabolic activities because the uptake of citrate by vacuoles may be limited by the low activity of the citrate transporters in the tonoplast [35].

Proteomic analyses are becoming useful options for the efficient identification of the genes controlling important agronomic traits. In a recent investigation of the genetic diversity associated with the organic acid concentration in apple germplasm, we observed that the mature fruits of two *Malus* species, *M. sargentii* and *M. niedzwetzkyana*, differ regarding their malic and citric acid contents [29]. In the current study, we analyzed the proteomes of the mature fruits of *M. sargentii* and *M. niedzwetzkyana*. A total of 31 proteins related to the metabolism of soluble sugars and organic acids as well as H^+^-ATPase were identified. Our findings will facilitate the characterization of the complex gene network regulating the soluble sugar and organic acid contents in apple fruits.

## 2. Results

### 2.1. Variation in Organic Acid and Soluble Sugar Contents in the Mature Fruits of Two Wild Apple Species

**Figure 1:**
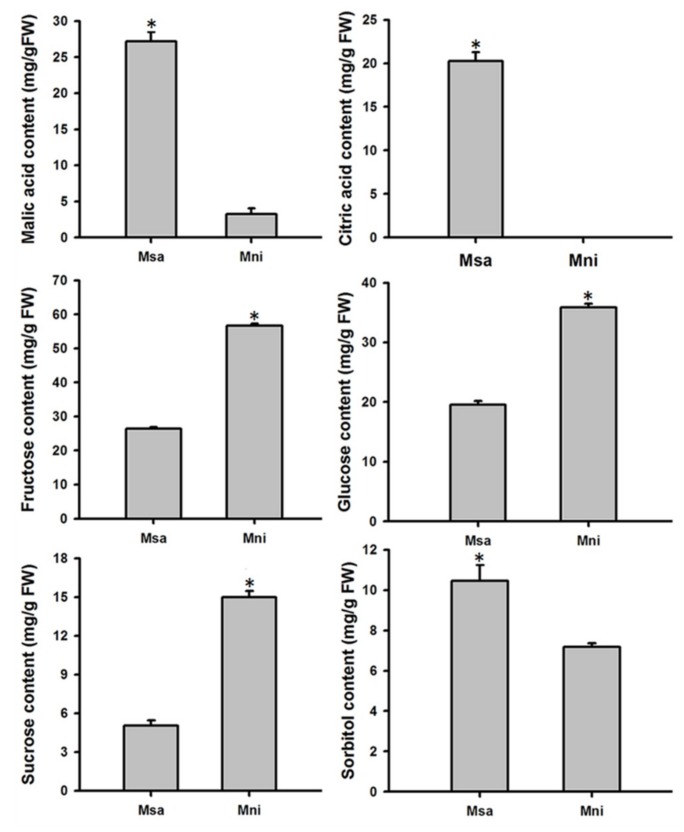
Variation of organic acid and soluble sugar content at maturity of two wide apple fruit. Msa: *M. sargentii*; Mni: *M. niedzwetzkyana*. The asterisks (*) indicate significant differences among genotypes (*p* < 0.01). The values represent the average of three biological replicates, and error bars show the SD of the mean. FW—fresh weight.

The organic acid (malic and citric acids) and soluble sugar (sucrose, fructose, glucose, and sorbitol) contents in the mature fruits of two wild apple species, *M. sargentii* and *M. niedzwetzkyana*, were determined with a high-performance liquid chromatography (HPLC) system (Figure 1). There were significant variations in the organic acid and soluble sugar contents between the mature fruits of *M. sargentii* and *M. niedzwetzkyana*. Regarding the organic acid content, a higher malic acid concentration was detected in *M. sargentii* mature fruits (27.20 mg/g fresh weight (FW)) than in *M. niedzwetzkyana* mature fruits (3.73 mg/g FW). Additionally, citric acid was detected only in *M. sargentii*, with an average concentration of 20.22 mg/g FW. An analysis of the soluble sugars revealed low fructose, glucose, and sucrose concentrations in *M. sargentii* mature fruits, with an average of 26.36 mg/g FW, 19.53 mg/g FW, and 5.06 mg/g FW, respectively. The sorbitol content was greater in *M. sargentii* mature fruits (10.44 mg/g FW) than in *M. niedzwetzkyana* mature fruits (7.18 mg/g FW). Notably, the average fructose and glucose concentrations were more than two times higher than the sucrose and sorbitol concentrations, implying fructose and glucose are the major soluble sugars in *M. sargentii* and *M. niedzwetzkyana* mature fruits.

### 2.2. Quality and Coverage of the Apple Fruit Proteome

In this study, procedures for the enzymatic hydrolysis of proteins, peptide quantification, and MS were completed to identify peptides, quantify proteins, and analyze differentially-expressed proteins. A total of 13,036 unique peptides were identified and 2901 proteins were quantified, with 2088 and 2347 identified in *M. sargentii* and *M. niedzwetzkyana* mature fruits, respectively (Table 1). Of the quantified proteins, 1697 (58.50%) were identified in both *M. sargentii* and *M. niedzwetzkyana* mature fruits. Moreover, 72.84% of the proteins were detected with two or more peptides, indicating the proteins were effectively separated and identified based on label-free shot-gun technology. The identification of proteins was highly reproducible across biological replicates, with 67.15% of all identifications reproducible in all three biological replicates, whereas 15.33% and 17.52% of the identifications were made in only two and one replicate, respectively.

Differentially-expressed proteins were defined as those whose abundance differed by more than one fold between the *M. sargentii* and *M. niedzwetzkyana* mature fruits (*p* < 0.05). A total of 1,079 differentially-expressed proteins were identified, of which 416 and 663 were more and less abundant, respectively, in *M. sargentii* mature fruits than in *M. niedzwetzkyana* mature fruits. Of these 416 and 663 proteins, 175 and 418 were exclusive to the *M. sargentii* and *M. niedzwetzkyana* mature fruits, respectively.

### 2.3. Verification of the Differentially Expressed Proteins by PRM

In this study, five candidate proteins were randomly selected and analyzed by LC-PRM/MS to verify the accuracy of the identification of differentially-expressed proteins (Table 2). The PRM results were consistent with the results of the label-free technique for the five analyzed proteins (MDP0000271088, MDP0000152497, MDP0000326249, MDP0000777702, and MDP0000308185), implying that the label-free LC-MS/MS proteomic analysis produced accurate and reliable quantitative data.

### 2.4. Analysis of Differentially-Expressed Proteins Based on the gene ontology (GO) and KEGG Databases

**Figure 2:**
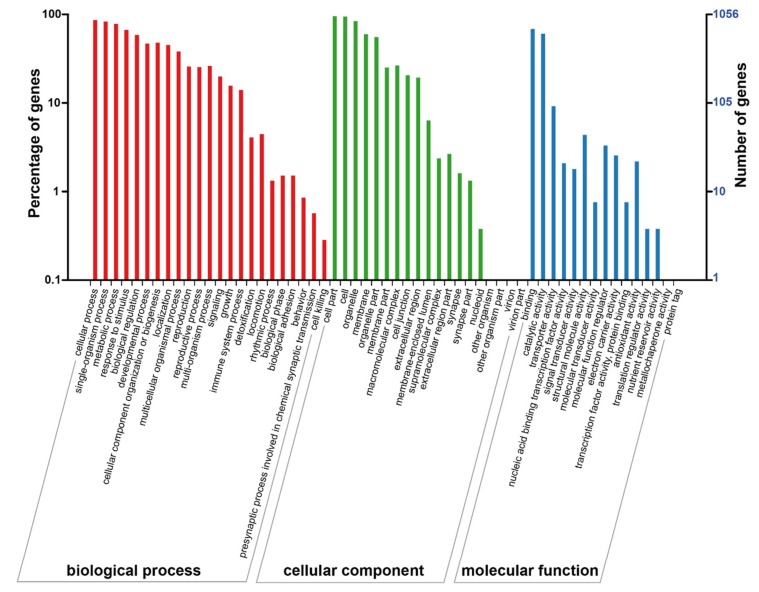
Gene ontology (GO) functional analysis results for the differentially-expressed proteins in mature fruits between *M. sargentii* and *M. niedzwetzkyana*.

The proteins that were differentially expressed between the *M. sargentii* and *M. niedzwetzkyana* mature fruits were functionally annotated based on the following three main gene ontology (GO) categories: biological process, cellular component, and molecular function [36,37] (Figure 2, Table S1). The results indicated that the significantly-enriched biological process GO terms were mainly related to nine processes (cellular process, GO:0009987; single-organism process, GO:0044699; metabolic process, GO:0008152; response to stimulus, GO:0050896; biological regulation, GO:0065007; developmental process, GO:0032502; cellular component organization or biogenesis, GO:0071840; localization, GO:0051179; and multicellular organismal process, GO:0032501). The significantly enriched cellular component GO terms were cell part (GO:0044464), cell (GO:0005623), organelle (GO:0043226), membrane (GO:0016020), and organelle part (GO:0044422). Finally, within the molecular function category, binding (GO:0005488) and catalytic activity (GO:0003824) were the main enriched GO terms.

**Figure 3:**
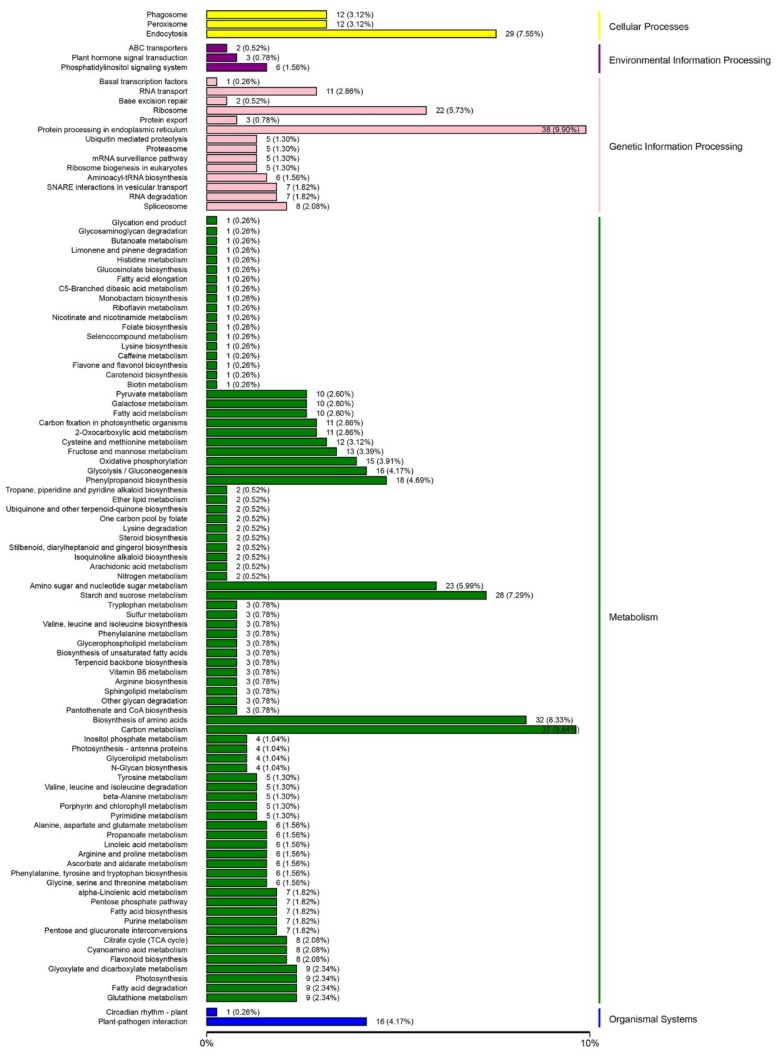
Kyoto Encyclopedia of Genes and Genomes (KEGG) pathways with the differentially-expressed proteins in mature fruits between *M. sargentii* and *M. niedzwetzkyana*. Each bar is followed by the number of the differentially-expressed proteins and its percentage.

The differentially-expressed proteins were classified into the following five groups based on the functional annotation with the KEGG database: cellular processes, environmental information processing, genetic information processing, metabolism, and organismal systems (Figure 3, Table S2). The KEGG classification results suggested the differentially-expressed proteins were mainly involved in metabolic processes. The metabolic pathways associated with the most differentially-expressed proteins were carbon metabolism (KO01200, Figure S1), biosynthesis of amino acids (KO01230), starch and sucrose metabolism (KO00500, Figure S2), and amino sugar and nucleotide sugar metabolism (KO00520). Carbon, starch, and sucrose metabolism are related to the conversion between primary metabolites, including soluble sugars and organic acids. Thus, through a series of reactions, soluble sugars, and organic acids can be efficiently synthesized and maintained at a certain concentration.

### 2.5. Specificity of the Protein Accumulation Related to Soluble Sugar and Organic Acid Metabolism

**Figure 4:**
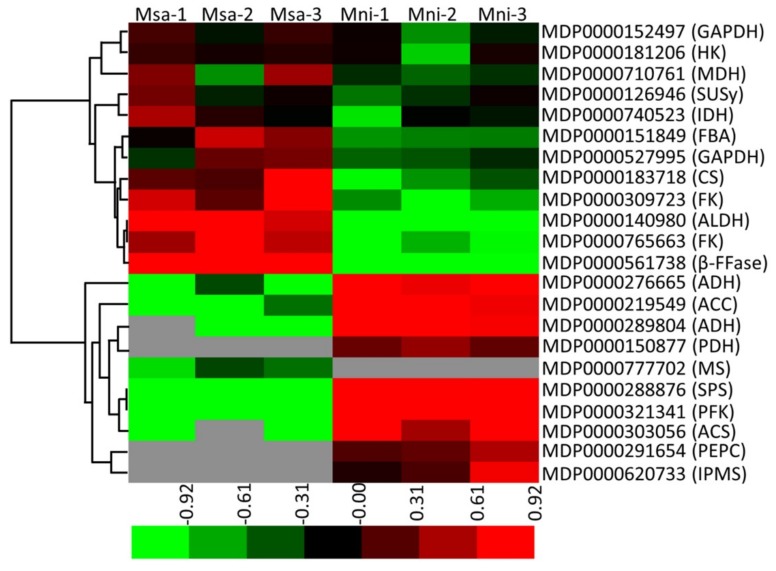
The differentially-expressed proteins involved in soluble sugar and organic acid metabolism in two *Malus* species mature fruits. The heat map was constructed using Mev software based on relative levels of differentially-expressed proteins, and normalized log^2^-transformed values were used to perform hierarchical clustering. Different colors represent differentially-expressed protein levels. MS: malate synthase; β-FFase: beta-fructofuranosidase; ALDH: aldehyde dehydrogenase; FK: fructokinase; CS: citrate synthase; FBA: fructose-bisphosphate aldolase; IDH: isocitrate dehydrogenase (NAD+); MDH: malate dehydrogenase; GAPDH: glyceraldehyde-3-phosphate dehydrogenase; SuSy: sucrose synthase; GAPDH: glyceraldehyde-3-phosphate dehydrogenase; HK: hexokinase; SPS: sucrose-phosphate synthase; ADH: alcohol dehydrogenase (NADP+); ACS: acetyl-CoA synthetase; ACC: acetyl-CoA carboxylase carboxyl transferase; PFK: phosphofructokinase; PEPC: phosphoenolpyruvate carboxylase; IPMS: 2-isopropylmalate synthase; PDH: pyruvate dehydrogenase.

The final organic acid and soluble sugar contents of mature fruits depend on their synthesis, degradation, and compartmentalization in subcellular organelles. To explore the mechanisms underlying the metabolism of soluble sugars and organic acids in *M. sargentii* and *M. niedzwetzkyana* mature fruits, the associated differentially-expressed proteins were identified. A total of 22 differentially-expressed proteins with known roles in the metabolism of soluble sugars and organic acids were identified (Figure 4, Table S3), including six enzymes of the Suc–Suc cycle (previously called ‘futile recycles’ [30]), five enzymes related to glycolysis, seven enzymes related to pyruvate metabolism, three enzymes of the TCA cycle, and one enzyme involved in the glyoxylate cycle.

Regarding the proteins involved in the Suc–Suc cycle, beta-fructofuranosidase (MDP0000561738), sucrose synthase (MDP0000126946), hexokinase (MDP0000181206), and two fructokinases (MDP0000765663 and MDP0000309723) were significantly more abundant in *M. sargentii* mature fruits than in *M. niedzwetzkyana* mature fruits. In contrast, the sucrose–phosphate synthase (MDP0000288876) level in *M. sargentii* mature fruits was approximately two fifths of that in *M. niedzwetzkyana* mature fruits. Among the proteins related to glycolysis, fructose-bisphosphate aldolase (MDP0000151849) and two glyceraldehyde-3-phosphate dehydrogenases (MDP0000152497 and MDP0000527995) were more abundant in *M. sargentii* mature fruits than in *M. niedzwetzkyana* mature fruits, whereas the abundance of phosphofructokinase (MDP0000321341), which is one of the rate-limiting enzymes of glycolysis, was much lower in *M. sargentii* mature fruits than in *M. niedzwetzkyana* mature fruits. Moreover, a phosphoenolpyruvate carboxylase (MDP0000291654) was identified only in *M. niedzwetzkyana* mature fruits. This enzyme is important for bypassing the reaction catalyzed by pyruvate kinase during glycolysis to mediate the synthesis of oxaloacetate (OAA) from phosphoenolpyruvate.

Pyruvate is a major product of glycolysis and is also an important intermediate during the transformation of sugars, organic acids, amino acids, and other compounds in fruits. Pyruvate dehydrogenase (MDP0000150877), which catalyzes the synthesis of acetyl-CoA from pyruvate, was detected only in *M. niedzwetzkyana* mature fruits. Aldehyde dehydrogenase functions downstream of the pyruvate metabolic pathway by converting acetaldehydes produced from pyruvate metabolism to acetate, which can then be reduced to acetyl-CoA by acetyl-CoA synthetase. The abundance of aldehyde dehydrogenase (MDP0000140980) in *M. sargentii* mature fruits was 9.25-fold higher than that in *M. niedzwetzkyana* mature fruits, whereas the acetyl-CoA synthetase (MDP0000303056) level was 41% lower in *M. sargentii* mature fruits than in *M. niedzwetzkyana* mature fruits. Moreover, two enzymes that help degrade acetyl-CoA (acetyl-CoA carboxylase carboxyl transferase (MDP0000219549) and a 2-isopropylmalate synthase (MDP0000620733)) were significantly less abundant (or undetectable) in *M. sargentii* mature fruits than in *M. niedzwetzkyana* mature fruits.

The TCA cycle is an important pathway involved in the conversion of di- and tri-carboxylates, including malate and citrate. Citrate synthase (CS), NAD-IDH, and NAD-dependent malate dehydrogenase (NAD-MDH) were the three enzymes involved in the TCA cycle that were identified in the present study. The CS (MDP0000183718) level was 4.05-fold higher in *M. sargentii* mature fruits than in *M. niedzwetzkyana* mature fruits (Figure 4, Table S3). Similarly, NAD-IDH (MDP0000740523), which degrades isocitrate to generate 2-oxoglutarate, was 2.79-fold more abundant in *M. sargentii* mature fruits than in *M. niedzwetzkyana* mature fruits. Additionally, NAD-MDH catalyzes the interconversion of OAA and malate, but it favors the synthesis of malate. The NAD-MDH (MDP0000710761) abundance was 2.64-fold higher in *M. sargentii* mature fruits than in *M. niedzwetzkyana* mature fruits. Furthermore, a malate synthase (MDP0000777702) associated with the glyoxylate cycle was identified, but only in *M. sargentii* mature fruits.

### 2.6. Identification of Proteins Involved in H^+^ transport

**Figure 5:**
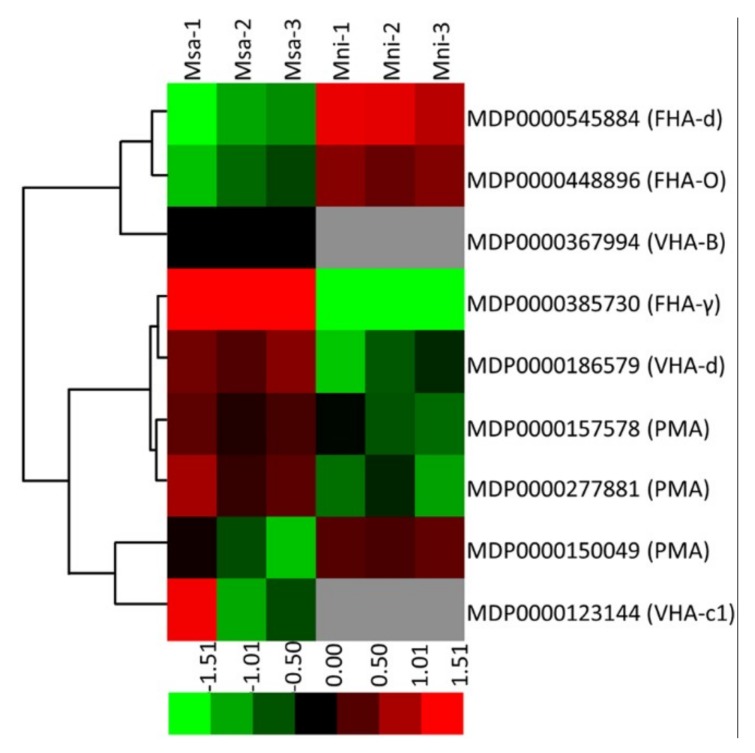
The differentially-expressed proteins involved in the H^+^ transport process in two *Malus* species mature fruits. The heat map was constructed using Mev software based on relative levels of differentially-expressed proteins, and normalized log^2^-transformed values were used to perform hierarchical clustering. Different colors represent differentiallyexpressed protein levels. VHA-c1: V-type H^+^-transporting ATPase subunit c1; VHA-B: V-type H^+^-transporting ATPase subunit B; FHA-γ: F-type H^+^-transporting ATPase subunit gamma; FHA-d: F-type H^+^-transporting ATPase subunit d; FHA-O: F-type H^+^-transporting ATPase subunit O; VHA-d: V-type H^+^-transporting ATPase subunit d; PMA: H^+^-transporting ATPase.

The H^+^-ATPases in plants catalyze the synthesis or hydrolysis of ATP coupled with proton translocation, and can be divided into the following three major classes based on structure, localization, and the mechanism underlying their function: F-type (mitochondrial or plastid membrane F_1_F_0_-ATP synthase), P-type (plasma membrane associated), and V-type (vacuolar or vesicular associated). Three F-type, three P-type, and three V-type H^+^-ATPases were identified in the current study (Figure 5, Table S3). The abundance of the three F-type H^+^-ATPases (MDP0000385730, MDP0000545884, and MDP0000448896) was significantly higher in *M. sargentii* mature fruits than in *M. niedzwetzkyana* mature fruits, whereas the abundance of the three P-type H^+^-ATPases (MDP0000157578, MDP0000277881, and MDP0000150049) exhibited the opposite pattern. Regarding the three V-type H^+^-ATPases, the abundance of VHA-d (MDP0000186579) was 2.02-fold higher in *M. sargentii* mature fruits than in *M. niedzwetzkyana* mature fruits, whereas VHA-c1 (MDP0000123144) and VHA-B (MDP0000367944) were detected only in *M. sargentii* mature fruits.

## 3. Discussion

Label-free proteomic techniques based on LC-MS/MS, which is widely used for quantitative analyses of protein expression, overcome the shortcomings of the traditional methods that cannot quantify proteins [19]. In plants, data derived from large-scale proteomic studies helped elucidate stress responses and tolerance [38,39], development [40,41,42], and metabolic fluxes and functions [30,43,44]. In the current study, a label-free proteomic analysis involving LC-MS/MS was performed to investigate the accumulation of soluble sugars and organic acids in the fruits of two wild apple species. The GO terms enriched among the 1,079 differentially-expressed proteins were mainly related to biological processes and cellular components. Additionally, the enriched KEGG pathways were primarily associated with metabolic processes, including carbon metabolism as well as starch and sucrose metabolism. Moreover, 31 of the differentially-expressed proteins were identified as involved in the metabolism of soluble sugars and organic acids as well as H^+^ transport. The LC-PRM/MS data for five differentially-expressed proteins verified the reliability of the label-free proteomic technique applied in this study.

**Figure 6:**
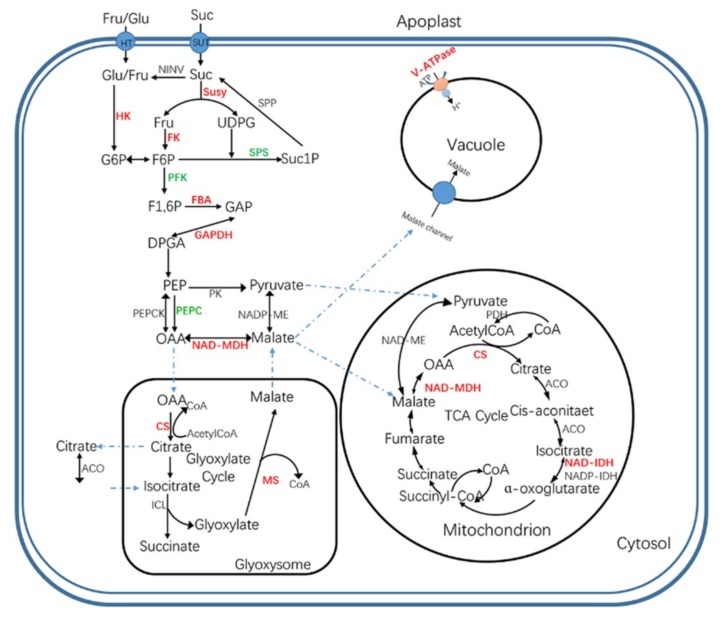
Metabolic network of soluble sugar and organic acid in apple fruits between *M. sargentii* and *M. niedzwetzkyana*. The red and green colors represent the proteins with up- and down-regulation in mature apple fruits in *M. sargentii* compared with that in *M. niedzwetzkyana*, respectively. For soluble sugar and organic acid metabolism: HK—hexokinase; FK—fructokinase; SUSY—sucrose synthase; SPS—sucrose-phosphate synthase; PFK—phosphofructokinase; FBA—fructose-bisphosphate aldolase; GAPDH—glyceraldehyde-3-phosphate dehydrogenase; PEPC—phosphoenolpyruvate carboxylase; NAD-MDH—NAD-malate dehydrogenase; CS—citrate synthase; MS—malate synthase; NAD-IDH—NAD-isocitrate dehydrogenase; Glu—glucose; Fru—fructose; Suc—sucrose; F6P—fructose-6-phosphate; G6P—glucose-6-phosphate; F1,6P—Fructose 1,6 diphosphate; GAP—glyceraldehyde 3-phosphate; DPGA—1,3-diphosphoglycerate; PEP—phosphoenolpyruvate; OAA—oxaloacetate.

In the current study, the soluble sugar and organic acid contents in the mature fruits of two *Malus* species, *M. sargentii* and *M. niedzwetzkyana*, were measured by HPLC. The major soluble sugars were identified as fructose and glucose, whereas malic and citric acids were detected as the two predominant organic acids, which is consistent with the findings of previous studies [2,29]. Soluble sugars, especially sucrose, glucose, and fructose, are responsible for the sweetness of fruits, whereas organic acids, such as malic and citric acids, determine fruit acidity. A moderate concentration of organic acids can increase the palatability of fruits, but low or high concentrations can decrease fruit quality [45,46]. In developing fruits, changes to the sugar and organic acid contents over time are due to the synthesis, degradation, and transport of these compounds. In the current study, 22 differentially-expressed proteins related to the Suc–Suc and TCA cycles were identified (Figure 6, Table S3). Sucrose synthase catalyzes the reversible conversion of sucrose to fructose and uridine diphosphate glucose. The resulting fructose can be phosphorylated by fructokinase to generate fructose-6-phoshate. Additionally, glucose can be phosphorylated by hexokinase to produce glucose-6-phoshate [47]. Thus, fructokinase and hexokinase play crucial roles in sugar metabolism and homeostasis. In our study, these three enzymes were more abundant in *M. sargentii* mature fruits than in *M. niedzwetzkyana* mature fruits, which may explain the high fructose and glucose contents in *M. niedzwetzkyana* mature fruits (Figure 1). Sucrose phosphate synthase is believed to be the key enzyme for controlling sucrose biosynthesis. It catalyzes the reaction in the sucrose synthesis pathway that produces sucrose-6-phosphate from fructose-6-phosphate and uridine diphosphate glucose (Figure 6). We detected a greater abundance of sucrose phosphate synthase (MDP0000288876) in *M. niedzwetzkyana* mature fruits than in *M. sargentii* mature fruits, which is consistent with the low sucrose content of *M. sargentii* mature fruits.

Fruit acidity is mainly due to the presence of malic and citric acids, which accumulate in fruit cells because of several interlinked processes that appear to be mainly under the control of genetic factors [32]. In fruit cells, malic acid is primarily synthesized in the cytosol by NAD-MDH [48], which mainly catalyzes the reversible conversion of OAA to malate [31,49]. Yao et al. revealed that the overexpression of apple *MdMDH*, which encodes NAD-MDH, leads to increased malate levels, suggesting its direct involvement in malate synthesis. The overexpression of *MdMDH* also results in the up-regulated expression of several malate-related genes, implying MdMDH also indirectly affects malate accumulation [49]. In the present study, the NAD-MDH (MDP0000710761) content was higher in *M. sargentii* mature fruits than in *M. niedzwetzkyana* mature fruits. Additionally, the glyoxylate cycle may influence malate accumulation in young grape berries and ripening banana fruits [50,51]. In our study, a malate synthase (MDP0000777702), which contributes to malate synthesis in the glyoxylate cycle, was detected only in *M. sargentii* mature fruits. These results suggest that the genes related to malate synthesis are crucial for the accumulation of malic acid in fruit cells. Citric acid is produced via the TCA cycle in mitochondria, with mitochondrial citrate synthase as the crucial enzyme controlling citrate synthesis [33,34]. Furthermore, citrate accumulation is mainly controlled by metabolic activities, not the uptake of citrate by vacuoles [35]. In our study, the CS (MDP0000183718) abundance was considerably higher in *M. sargentii* mature fruits than in *M. niedzwetzkyana* mature fruits, indicating that CS likely controls the citric acid content in *M. sargentii* mature fruits.

Vacuoles are a major repository for organic acids, as evidenced by the fact that organic acid concentrations in the vacuole exceed the corresponding concentrations in the cytosol by several-fold [32,52]. In the cytosol, at neutral or slightly alkaline pH, almost all of the malate and citrate are in the form of dianions and trianions, respectively. These forms can be transported into vacuoles, wherein the dominant species are the protonated forms because of the acidic nature of vacuoles. After the dianions and trianions cross the tonoplast to reach the acidic vacuole, they are immediately protonated, which maintains their electrochemical potential gradient and enables their continuous transport into the vacuole (i.e., acid trap mechanism) [32,53]. The trapping efficiency depends on two factors, namely the vacuolar pH and the electric potential gradient across the tonoplast, which are generated by the protons pumped into the vacuole. The V-type H^+^-ATPases are one of the major proton pumps in fruit cell vacuoles [32]. They can hydrolyze the high-energy phosphate bond of ATP and promote the transport of protons into vacuoles. Several studies suggested that the difference in the organic acid content between fruit species and between different cultivars of the same fruit species may be linked to the diversity in their proton pumps [10,54,55]. In the present study, three V-type H^+^-ATPases were more abundant in *M. sargentii* mature fruits than in *M. niedzwetzkyana* mature fruits. Thus, we speculate that the higher malic and citric acid contents in *M. sargentii* mature fruits than in *M. niedzwetzkyana* mature fruits are likely due to the greater ability of *M. sargentii* mature fruits to synthesize malic and citric acids as well as the higher trapping efficiency of their vacuoles for malate and citrate.

## 4. Materials and Methods

### 4.1. Plant Materials

The two *Malus* species used in this study, *M. sargentii* and *M. niedzwetzkyana*, were grown at the Horticultural Experimental Station of Northwest A&F University, Yangling, Shaanxi province, China, with standard horticultural practices and pest and disease control measures. In 2016, mature fruits were collected at 90 days after full bloom. The maturity of the collected fruits was confirmed based on their weight, seed color, and the results of a starch iodine test. Fruits with a uniform size and color and free of visible injuries or blemishes were selected for the subsequent experiments, which were completed with at least three biological replicates. Five fruits sampled from the same tree constituted one biological replicate. Pooled fruit samples were peeled, sliced into pieces, transferred to a pre-chilled centrifuge tube, flash-frozen in liquid nitrogen, and stored at −80 °C until analyzed.

### 4.2. Determination of Soluble Sugar and Organic Acid Contents

The soluble sugar and organic acid contents in apple fruits were measured with an HPLC system as previously described [2]. Briefly, for each replicate, approximately 5 g of fruit sample were added to liquid nitrogen in a mortar and ground to a powder with a pestle. Next, 1 g of ground powder was dissolved in 5 mL ddH_2_O from the Milli-Q Element water purification system (Millipore, Bedford, MA, USA). The mixture was incubated for 30 min in a water bath set at 37 °C prior to an ultrasonic extraction at room temperature for 15 min. The resulting solution was centrifuged at 5000× *g* for 15 min at 4 °C. The supernatant was passed through a 0.22 μm Sep-Pak filter (ANPEL, Shanghai, China) and then analyzed with the Agilent 1260 Infinity HPLC system (Milford, MA, USA) to measure the soluble sugar and organic acid contents.

To detect the organic acids, the HPLC system was coupled to a diode array detector set at 210 nm. The chromatographic separation was conducted on an Athena C18 column (4.6 × 250 mm, 5 μm). Specifically, the column temperature was maintained at 40 °C, the mobile phase was 0.02 M KH_2_PO_4_ solution (pH 2.4), the injection volume was 20.0 μL, and the flow rate was 0.8 mL/min.

The soluble sugars were detected with the HPLC system coupled to a refractive index detector with the reference cell maintained at 90 °C. The chromatographic separation was conducted with a Transgenomic COREGET-87C column (7.8 mm × 300 mm, 10 μm) and a Transgenomic CARB Sep Coregel 87C guard column cartridge. The mobile phase was ddH_2_O and the flow rate was 0.5 mL/min. The soluble sugar and organic acid standards used during the HPLC analyses were purchased from Sigma (St. Louis, MO, USA) and dissolved in deionized water.

### 4.3. Protein Extraction, Enzymatic Hydrolysis, and Peptide Quantification

Fruit samples were frozen in liquid nitrogen and ground with a mortar and pestle. The powder was resuspended in five volumes of TCA/acetone (1:9) and vortexed. The mixture was incubated at −20 °C for 4 h and then centrifuged at 6000× *g* for 40 min at 4 °C. The supernatant was discarded and the pellet was washed three times with pre-cooled acetone. The precipitate was air-dried. Thirty volumes of SDS with DTT (SDT) lysis buffer (4% SDS, 100 mM dithiothreitol (DTT), and 150 mM Tris-HCl, pH 8.0) was added to 20–30 mg dried sample and the resulting mixture was boiled for 5 min. The lysate was sonicated and then boiled for 15 min. After centrifugation at 14,000× *g* for 40 min, the supernatant was passed through a 0.22 µm filter and stored at −80 °C until analyzed.

The samples were digested with a tryptic solution according to a modified version of an established filter-aided sample preparation protocol [56]. All of the required chemicals were purchased from Sigma, unless otherwise stated. For each sample, 200 μg protein were mixed with 30 μL SDT buffer. The DTT and other low-molecular-weight components were removed with UA buffer (8 M urea and 150 mM Tris-HCl, pH 8.0) and repeated ultrafiltration. Next, 100 μL iodoacetamide (100 mM in UA buffer) were added to block reduced cysteine residues and the samples were incubated for 30 min in darkness. The filters were washed three times with 100 μL UA buffer and then twice with 100 μL 25mM NH_4_HCO_3_ buffer. Finally, the protein suspensions were digested with 4 μg trypsin (Promega) in 40 μL 25 mM NH_4_HCO_3_ buffer overnight at 37 °C, and the resulting peptides were collected as a filtrate. The peptides of each sample were desalted with Empore™ SPE Cartridges (C18; standard density, 7 mm bed I.D., and 3 mL volume; Sigma) and then concentrated by vacuum centrifugation and reconstituted in 40 µL 0.1% (v/v) formic acid.

The LC-MS/MS analysis (120 min) was performed with the EASY-nLC system (Thermo Fisher Scientific) coupled to the Q Exactive mass spectrometer (Thermo Fisher Scientific), which was operated in the positive ion mode. The MS data were acquired with a data-dependent top-10 method that selected the most abundant precursor ions from the survey scan (300–1800 m/z) for HCD fragmentation. The automatic gain control target was set to 3e^6^ and the maximum injection time was set to 10 ms. The dynamic exclusion duration was 40.0 s. Survey scans were acquired at a resolution of 70,000 at m/z 200. The resolution for the HCD spectra was set to 17,500 at m/z 200 and the isolation width was m/z 2. The normalized collision energy was 30 eV and the underfill ratio (i.e., the minimum percentage of the target value likely to be reached at the maximum fill time) was defined as 0.1%.

### 4.4. Data Analysis

The original raw LC-MS/MS data were analyzed with the MaxQuant software (version 5.3.17) to screen databases [57]. The main library search parameters are listed in Table 3.

### 4.5. Bioinformatic Analysis

The differentially-expressed proteins in the mature fruits of two wild apple species underwent a bioinformatic analysis. The Blast2GO program (http://geneontology.org/) was used to functionally annotate the differentially-expressed proteins. Additionally, the identified proteins were classified and grouped based on the KEGG database (http://www.genome.jp/kegg/). The significance of each identified enriched pathway and GO term were determined with Fisher’s exact test, and the target proteins were analyzed based on the GO terms and KEGG pathways [37,58].

### 4.6. PRM Verification

The LC-PRM/MS analysis was conducted with an HPLC liquid phase system Easy-nLC1200 (Thermo Fisher Scientific, Waltham, USA) as previously described with minor modifications [36]. The relevant liquid phase gradient was as follows: 0–2 min, with a linear gradient of B liquid from 5%–10%; 2–45 min, with a linear gradient of B liquid from 10%–30%; 45–55 min, with a linear gradient of B liquid from 30%–100%; 55–60 min, with B liquid maintained at 100%. The peptides were chromatographed and analyzed using a Q-Exactive HF Mass Spectrometer (Thermo Scientific). Analysis duration: 60 min. Detection method: positive ion. Parent ion scanning range: 300–1800 m/z. The mass-to-charge ratio of peptide and polypeptide fragments was collected as follows: 20 fragment maps (MS2 scan); mass spectrometry resolution: 30,000 (@ m/z 200); AGC target: 3e^6^; maximum IT: 120 ms; MS2 activation type: HCD; isolation; normalized collision energy: 27. 

## Figures and Tables

**Table 1:** Statistics on the protein identification results.

Identification Results	Unique Peptides	Quantified Proteins	Msa vs. Mni		Significantly Different Proteins
			Up-Regulated	Down-Regulated	
Total	13036	2901	416	663	1079

**Table 2:** Quantitative results for five candidate proteins determined using the parallel reaction monitoring (PRM) methods.

Peptide Sequence	Protein Name	Average Content		PRM Fold Change	Label-Free Fold Change	Consistency between PRM and Label-Free
		Msa	Mni			
EYYTNALAAAK	MDP0000271088	0.0127	0.1152	0.1106	0.7260	Yes
VSLGNFPDLAGAVNK						
FLVSDSFPGNDR	MDP0000152497	0.6065	0.7086	0.8559	0.3358	Yes
LVPIINPTTR						
INDQAGYSSFR	MDP0000326249	0.4397	0.0483	9.1035	2.9726	Yes
LANILHANELAR						
ANEAALDLVR	MDP0000777702	0.3039	/	_	_	Yes
YNEGALPGFDPATK						
AFVDSGAQSTIISK	MDP0000308185	/	0.2294	_	_	Yes
GIAHGVGQSEILGR						
LVELGFGR						

**Table 3:** The MaxQuant search library parameter settings.

Item	Value
Enzyme	Trypsin
Max missed cleavages	2
Max missed cleavages	2
Main search	6 ppm
First search	20 ppm
MS/MS tolerance	20 ppm
Fixed modifications	Carbamidomethyl (C)
Variable modifications	Oxidation (M), acetyl (protein N-term)
Peptide FDR	≤0.01
Protein FDR	≤0.01
Time window (match between runs)	2 min
Protein quantification	Razor and unique peptides were used for protein quantification.

## Data Availability

The raw data is currently being prepared for submitting to Proteome Xchange (http://www.proteomexchange.org/).

## Supplementary Materials

The following are available online at https://www.mdpi.com/2223-7747/8/11/488/s1. Figure S1: KEGG map of carbon metabolism pathway based on the differentially-expressed proteins; Figure S2: KEGG map of starch and sucrose metabolism pathway based on the differentially-expressed proteins; Table S1: GO (gene ontology) functional analysis results for the differentially-expressed proteins in mature fruits between *M. sargentii* and *M. niedzwetzkyana*; Table S2: KEGG (Kyoto Encyclopedia of Genes and Genomes) pathways with the differentially-expressed proteins in mature fruits between *M. sargentii* and *M. niedzwetzkyana*; Table S3: the potential key proteins involved in organic acid and soluble sugars metabolism in two *Malus* species.
